# Social Intervention and Governance of Youth School Bullying—Based on Computer Medical Data Analysis

**DOI:** 10.3389/fpubh.2022.881124

**Published:** 2022-05-20

**Authors:** Jiahui Zhao

**Affiliations:** School of Government, Shanghai University of Political Science and Law, Shanghai, China

**Keywords:** school bullying, teenagers, big data, social work, drama education, behavioral interventions

## Abstract

The rise of computational social science provides a new method for campus bullying research based on large-scale data collection, calculation and analysis. Governing the bullying behavior of a middle school through social intervention, and closely observe the service needs and existing problems of the school youth group. This paper analyzes the characteristics, inducements and negative effects of school bullying. Combine drama courses and working group education methods to intervene in school bullying. Intervention work includes making teenagers aware of bullying behavior and identifying bullying types. To achieve the purpose of empathy through role play, bullies can effectively control irrational thoughts, understand their own cognitive biases, and reconcile their own emotions and behaviors. So that the victims can identify the bullying behavior around them in time, and cultivate their resistance and self-protection awareness in the event of bullying. Based on the empirical analysis of social work to intervene in the practical dilemma, and put forward the corresponding countermeasures to reduce the negative impact of school bullying on all aspects of youth, so as to reduce the various social risks brought by school bullying.

## Introduction

At present, campus bullying is common. Campus bullying is a kind of behavior that intentionally injures the personal and property safety of others, including beating, intimidating, cursing, isolating, controlling, insulting, laughing and so on. Rodríguez-Muoz et al. (1) investigated the phenomenon of bullying in local primary and secondary schools. After in-depth analysis, he believes that intimidation is the main manifestation of bullying behavior among teenagers in school. Bullying is a behavior that continuously harms others. Due to the imbalance of social and physical and mental strength between the victim and the abuser, the victim suffers long-term and continuous harm in the process of bullying (1, 2).

In terms of school bullying (2), stated that bullying means that “in the same group, the powerful party uses words and behaviors to intentionally bring physical and mental stress and harm to the vulnerable group members”. Japan's education ministry in the early 1990s defined bullying as “unilateral, sustained physical and mental attacks that can cause great pain to others”.

Park et al. (3) points out that “Bullying is a pervasive aggressive behavior. This behavior is often characterized by threatening or intimidating others.” Webb et al. (4) believes that school bullying refers to the intentional and violent assault, including physical and psychological assault, on others by means of beating, harassment, isolation and other means. According to Ran et al. (5), campus bullying refers to the continuous bullying of the strong against the weak and the large group against the small group in the campus, which is in conflict with or directly related to the high school students (3, 4).

According to the above literature description, it is not difficult to find that although there is no unified definition of school bullying, it can directly or indirectly reflect the asymmetry of power between the bully and the bullied. Carreira et al. (6) believes that bullying is a common social phenomenon with the characteristics of concealment, diversity, repetition, imbalance, universality and indistinguishable (5). Georgiou et al. (7) believes that bullying is characterized by diverse forms, violent behavior, fragile object and extensive influence (8).

To effective governance adolescent bullying behavior, improve the adolescent bullying phenomenon, at the middle school has carried on the empirical research, this paper adopts the social work intervention combined with drama class, effectively improve the middle school campus bullying problems, and analyze the intervention of social work practice difficulties, some countermeasures are put forward. Finally, it provides references for enriching bullying-related theories. The research on campus bullying in my country started late and the pace is slow, the literature is not concentrated and the understanding is not comprehensive, and most of them are analyzed and studied from the perspective of psychology and sociology. This research adopts practical investigation, analyzes the situation and influencing factors of the problem with the help of theoretical tools, and tries to put forward strategies of management, implementation and guarantee for the prevention and treatment of school bullying from the perspective of pedagogy theoretical research.

## Student Group Needs and Social Work Intervention Methods

### The Needs of Student Groups in the Context of School Bullying

In recent years, the incidents of campus violence have been frequent, which has aroused widespread concern in the society (9, 10). The Office of the Education Supervisory Commission of the State Council issued a special action plan on cracking down on school bullying in May 2018, calling for a severe crackdown on intentional intimidation and bullying by primary and middle school students using physical, verbal and online technology. In addition, a survey on the harmonious situation of teenagers in 10 middle schools in a certain area was conducted. The self-reported bullying rate was 22.3%, the self-tested bullying rate was 13.8 and 33.5% of students said they had witnessed bullying, indicating that it was more serious (11, 12). School bullies seem to be everywhere, and they have different degrees of influence on the growth of teenagers. Even the “mass psychology” of bullying leads children to use similar solutions and imitate the bully to behave in unsafe ways. It threatens the physical and mental development of primary and middle school students. On this basis, the author believes that it is necessary to teach the bully and the victim to express their thoughts from the perspective of emotional control, irrational expression and control, replace violence with correct expression, help the bully and the victim to establish a good relationship, and ultimately reduce the occurrence of bullying (13, 14).

Working at a social workstation in a middle school, I got to know the details of the middle school as follows: Grade 7 was divided into 6 classes with 260 students, Grade 8 was divided into 8 classes with 340 students, Grade 9 was divided into 8 classes with 240 students. The school provides educational resources not only for registered students, but also for children of migrant workers with excellent academic performance. So the students come from different sources (15, 16). The ratio of family enrolments to migrant students is currently about 6:4. In addition, classes will be arranged for students, including those with learning disabilities and disabilities, in a number of classrooms.

Through questionnaires and observations, social workers have found that different groups in schools will need different services by 2019–2020. Group service and demand analysis are shown in Tables 1, 2.

**Table 1:** Group services and demand analysis.

**Group**	**Demand**
Seventh grade students	1. Environmental adaptation needs: freshmen in the seventh grade cannot adapt to the middle school life due to the changes in learning content, work and rest rules and campus environment.
	2. Running-in needs for interpersonal communication: Because teenagers are in a special stage of growth and development, belonging to the running in period of self and society, their hearts are more sensitive, it is difficult to integrate into the collective and establish a stable interpersonal relationship with classmates.
	3. Adolescent attention needs: Grade 7 students are in a critical period of physical and psychological development, which can also be called the rebellious period. During this period, great changes occur in body and mind, which requires more attention from parents and teachers.
Eighth grade students	1. Running-in needs of interpersonal communication: Grade 8 students have not left the growth and development period of teenagers, so it is difficult for them to integrate into the group and establish a stable interpersonal relationship with their classmates, and some bullying behaviors may occur.
	2. Adolescent Guidance Needs: Grade 8 students have adapted to their physical and mental changes during adolescence. However, due to their ignorance of the opposite sex, they tend to have puppy love or stay away from the opposite sex.
	3. Learning objective planning needs: Due to the physical and mental changes, the eighth grade students' attention is easy to be shifted, it is difficult to focus on learning, lack of learning goals and learning motivation, so they can not keep up with the curriculum, resulting in inferiority complex.
Ninth grade students	Planning of learning direction: Grade 9 students have initially formed an independent personality. Due to the change of puberty, students are focusing on interpersonal relationship, which leads to their loss of learning goals and motivation, and they have no specific plan for future study.

**Table 2:** Special group services and needs analysis.

**Group**	**Demand**
Students	1. Adapting Habits: As the student still has the habits brought by the former campus and interpersonal environment, these habits may be in conflict with the habits of the students in the current campus, such as hygiene habits, work and rest rules, dialect, etc., which hinders the student's integration and adaptation in the new environment.
	2. The need to enhance family attention: due to the changes of study and interpersonal environment, students are more sensitive and prone to inferiority, so parents need to pay more attention to the changes.
	3. The need for acceptance among classmates: due to the changes in the new environment, students hope to make new friends as soon as possible and keep up with the new pace of study, so they need to be accepted by classmates.
Poor students	1. Increase the need for self-confidence: due to the lack of economic conditions, poor students are very likely to have a sense of inferiority, or extreme conceit.
	2. Material needs: Compared with other types of student groups, poor students lack material conditions more. They need basic material conditions from their families to reduce the gap between them and their classmates.
Students with different behaviors	1. Constraint and guidance needs: Because parents and teachers do not pay attention to students' different behaviors, students do not know that some of their behaviors are wrong, resulting in students' cognitive and behavioral bias.
	2. The need to be tolerated: because their individual behaviors are not understood, other students will label them, and even discriminate against and misinterpret them. Therefore, such students need to be treated with tolerance.

This suggests that eighth-graders, students on loan and students with different behaviors are more likely to have school bullying (17, 18). Without further research, the author reached a consensus with the school to organize eighth grade students to participate in street campus bullying exhibition and campus bullying prevention drama working group, so as to help control campus bullying and create a harmonious campus.

Combined with the results of the unstructured interview, it is concluded that there are both bullies and victims in the working group. Most of them have four problems. Problem 1: Bullies, victims, and bystanders do not recognize that some of the actions they are dealing with are bullying, and do not know how to deal with bullying. Problem 2: At the family level, there are problems such as poor family communication, inadequate family structure, lack of care or inappropriate parenting methods, which are all factors that lead to violent behavior. Bullies often believe that their parents do not understand them, there is a lack of communication and a generation gap. The families of the victims lack communication, they don't like to talk at home, so the parents can't observe the inner changes of the children. Problem 3: In school, teachers tend to pay more attention to students who are good in grades and obedient. Bullies often think that teachers always take themselves as targets or negative teaching materials, so they don't listen to the teacher's orders, and arouse the teacher's attention and recognition by singing the opposite. Problem four: in interpersonal relationships, bullies and victims cannot find intimate friends, they are low self-esteem, lack of confidence. Cowardly and cowardly students are often bullied and ostracized; Students who bully others often hope to establish their status through violence. Bullies believe that power makes people obedient, afraid, safe, and valued. So, the members of the task force have several requirements: First, be aware of bullying. Secondly, it is necessary to understand interpersonal relationships and interpersonal communication skills. The second is to learn how to prevent school bullying and how to protect yourself when it happens (19, 20).

### Purpose and Method of Social Work Intervention

Recruit bullies and victims of bullying and use professional social work tools to intervene in bullying and violence incidents. To understand the psychological needs of students, give appropriate treatment, so as to achieve better results, improve the extreme psychology of bullies, so that they reduce aggressive behavior. Develop the victim's confidence in the outside world so that he or she can seek help when confronted with bullying problems (21, 22). A good campus environment helps to correct students' personal behavior, so the construction of a “harmonious campus” environment can enable students to receive good education, so that students have normal interpersonal relations, conducive to the overall development of body and mind.

The use of social work intervention to deal with campus bullying is the main way of social work. The form of social work intervention can promote the working group and its members to achieve their needs, so that students can accelerate their own social transformation through working group. Coordinate and develop interpersonal relationships between individuals and working groups; So as to play the social functions of organizations and groups, improve the social psychology of bullying and victims, so that they can get a sense of social identity and belonging. After establishing a clear goal, team members can help each other and collectively obtain more resources. It makes the members of the working group actively communicate, understand each other, have the courage to take responsibility and get treatment in the mutual cooperation, so as to promote positive changes in the attitude and behavior of the team members (23, 24).

The working group always adopts the way of role play. Role-playing is to understand a variety of behavior habits and norms in the society by playing the role of others, learn to understand the psychological activities and behavior content of others, and finally realize the social transformation of team members. By playing different interpersonal roles, we can gain insight into the positions and psychological activities of the roles we play. In the process of empathy, we can get a better way to deal with campus bullying and interpersonal relationship.

Drama courses are also the basis for social work interventions. Through the drama course, the working group realized the interpretation and correction of school bullying. Drama courses should not only learn the knowledge and interpretation skills of drama, but also enable students to understand the society, think about life, control behavior and protect themselves through drama. Drama course is to introduce drama activities into the field of education and use drama background to achieve educational purposes.

The purpose of drama course in social work intervention is to change the common phenomenon of campus bullying. So that the bully can understand the hurt of the bully through scene modeling, role playing and other methods, and make the bully brave to ask for help when hurt.

## Analysis of School Bullying Behavior

### Characteristics of School Bullying Behavior

Bullying is not an occasional argument or fight. It is an unfair behavior where one party constantly puts pressure on the other. Whether the bystander, the bully or the bullied, campus bullying will bring profound physical and mental harm. In recent years, there have been media reports of “a dispute case concerning the right to health accepted by a certain court”. Some junior high school girls beat up another girl, leaving her handicapped. The incident started when the victim did not pay attention to personal hygiene during school, which caused hostility from other female students in the dormitory. Five schoolgirls slapped her in the face to “educate” her, resulting in a grade 10 disability.

After finishing the junior high school Chinese test, 16-year-old Xiao Huang endured great pain and continued to take the senior high school entrance examination. For 4 years, he kept a secret from his parents: Starting in the fifth grade, he was regularly caught in “unprovoked attacks” by several other students. This kind of school bullying is not uncommon in the media.

A simple message can tell us that bullying is insidious, persistent and communal. School violence is frequent and hidden. Bullying often takes place out of the reach of teachers and parents. For example, dormitory and toilet, whose daily use is relatively private, it is hard to be found by others. Many victims have been bullied for years but do not have the courage to ask their parents and teachers for help. This cowardice makes the bully more aggressive, less moral, and more stubborn. School bullying has a fixed pattern, that is, victims are always bullied; Bullying is not an individual act. It occurs in small groups and groups. They have similar characters and imitate each other in their actions. Bullying behavior is a group behavior formed by a person under multiple individual pressures.

### Causes of School Bullying

Middle school students are in a critical stage of physical and mental development, they often do not have enough experience to deal with problems harmoniously, and they cannot empathize with others. So the way they deal with some of these problems is to emulate other students. Personality traits also play an important role in bullying behavior. Bullies generally have strong personalities and need external recognition and “face saving”. They believe that bullying can make them stronger and save face more. Bullies are introverted and timid. They dare not ask for help when being bullied. They can only swallow their pride and become the objects of long-term bullying. There are also some victims of bullying who have birth defects or bad habits. They are often mocked and insulted, but their innate condition makes them unable to resist.

Bullying is not only a personal problem for students, family relationships are also an important cause of school bullying. Every family has a different way of raising children. Some parents do not care about their children's life and psychological state, lack of guidance and education for children's growth, resulting in the lack of correct supervision and guidance of children's behavior, it is easy to lead to bullying behavior; In some families there is tension and parents often scold their children. This leads to a lack of security and the belief that violence is the only way to solve the problem. Lead to bullying behavior to solve conflicts with peers; In some families, the parents are very bossy and don't care about the inner needs of the children. They only demand obedience and obedience from the children. The child who receives this kind of family education will become weak, self-abased, unsociable, etc., and the child will often become the object of bullying. Therefore, the family relationship and parents' education style will decide the child's growth performance (25, 26).

The school environment, as the cradle of children's education and growth, is another factor that leads to bullying behavior. The strict management mechanism of the school will greatly restrict the bad behavior of the school, so that good communication between teachers and students is not only the responsibility of teachers, but also an important condition for students to grow up. To enhance teachers' sense of mission in education, make them pay attention to students' academic performance and psychological state, so as to reduce students' bullying behavior. Or when bullying behavior occurs, teachers can perform their duties, effectively curb the continuation of bullying behavior, communicate with parents in time, and do a good job in family work between the bully and the bullied, so as to solve the problem of bullying on campus from the root.

### The Negative Effects of School Bullying

The primary problem of school bullying is that the victim is physically bullied. These include beating, shoving and other acts that may cause physical injury to the victim. Because trauma is especially easy to spot, it's also easy for the media to see physical bullying. Because bullies fear that the outside world will find out about their bad behavior, they choose to beat their victims by avoiding obvious places, such as the face and limbs. Most of the victims have wounds in the abdomen and lower body, leaving internal wounds that are hard to see outside. The health of the victims has been greatly affected.

Moreover, the victims have suffered irreparable psychological trauma. Bullies have been abused, beaten and teased for a long time. Because of the imbalance of power, he will endure hidden psychological pressure and pain for a long time, which will lead to the formation of a pessimistic personality, negative, cowardly and fearful character, not only difficult to maintain their studies, they often fear in life, do not know how to live, do not know how to get along with others. Some victims of intolerance turn to extreme behaviors, such as suicide or self-harm, which can have a devastating effect on their mental health and development. Bullies, on the other hand, usually have poor grades and are “face-saving”, and they use violence to prove their authority. In addition to forming “cliques” among their peers, they also have difficulty communicating with others and often threaten vulnerable groups with violence. It is easy to break the law and crime after becoming an adult, which has a negative impact on social development.

## Social Work Intervention

### Working Group Intervention Objectives

The overall goal of the task force's intervention was to reduce the irrational thinking and behavior of bullying participants through drama lessons and role-playing, to develop a more comprehensive understanding of bullying, and to identify appropriate skills to deal with bullying. Establish good interpersonal relationship and promote campus harmony.

The phased objectives of the intervention by the working group are as follows: firstly, to improve the students' overall awareness of school bullying, so that the working group members can understand the concept, types, hazards and manifestations of school bullying. The second is to change the irrational thinking of bullies, break through the communication barriers between bullies and teachers, parents and classmates, learn interpersonal communication skills, and establish a good interpersonal relationship. The third is to enable the victims of bullying in the group to learn not to let others hurt them, let the bullies know that they have the ability to control their own aggressive behavior, and promote them to establish the values of helping others and harmonious progress together.

### Working Group Intervention

Working Group Name: “Peace Elite” Campus Drama Curriculum Working Group

Intervention period: 2:00–4:00 p.m. every Wednesday from March to June 2020

Number of activity classes: 6

Duration: 120 min per session

Group size: 6 people

(1) Section 1: Establishment of working group relationship. See Table 3 for details.
  The first drama course is the first step in the development of social work, marking the initial stage of social work intervention in the treatment of school bullying. Since the working group has just been set up and the members are not familiar with each other, the social workers will choose sand tools and sand paintings to let the members show themselves and get to know each other, and to create an atmosphere of mutual trust and ease in the group. Understand and interact with team members on their work goals and expectations to increase their participation and sense of belonging.
  Course summary: After the social worker introduced himself first, some team members were gradually led to introduce themselves, but some team members did not adapt to the team atmosphere, and their performance was relatively cold. Working groups should start with ice-breaking sessions so that members can interact more and have more opportunities to get to know each other. The members of the later working group have reached the stage of mutual understanding, and have a certain impetus to the working group, from which the cohesion gradually formed. After that, the communication and motivation between team members will be further strengthened through role playing.
(2) Section 2: How to identify school bullying. See Table 4 for details.
  Course Analysis: As the second stage of social work intervention, this course solves the problem of members' lack of participation and unity in the early stage of working group construction through the ice breaking link. At the same time, in order to make the team members know more about campus bullying, the working group should also be responsible for organizing the interaction and questioning among the team members, and explaining the relationship between drama course and campus bullying for the team members. It is also necessary for the members of the group of social work organizations to watch the film, share their impressions and encourage them to express their views through sharing. And in the process of watching the film, the freeze frame screen specifically analyzes the specific types of campus bullying, so that the students participating in the working group can better understand the knowledge of campus bullying. The second section of the course reduces the resistance of group members to the intervention of social work, and the internal vitality of the working group increases with the deepening of the course, gradually forming cohesion (27, 28).
  Course Summary: Compared with the first working group course, in the process of carrying out the second course, the team members have eliminated the formality and constraints. Therefore, compared with the first course, this course has been recognized by more team members, and the interaction within the group is gradually frequent. At the beginning of the course, participants were not very active, but when the social worker shared the meeting, participants were attracted; When watching the video, I was very focused. After watching the film, the group members can enthusiastically discuss its content. So the social worker must personally instruct the team members, prepare more cases to share with them, and use multimedia to attract them into the team. Second, members of the working group responded strongly to the video and could easily ask or answer other less relevant questions during the discussion. In this lesson, due to the divergent thinking of middle school students, it is necessary to bring the subject of the course back to the issue of school bullying in time when the course is off topic.
(3) Section 3 Working Group: To recognize their own irrational thoughts. See Table 5 for details.
  Course Analysis: The working group has entered the third session on intervention in school bullying. In this lesson, the focus is to understand the irrational thinking of the bully. To understand what irrational thinking is, let the group members find irrational thinking in their daily life. Further strengthening the contact and communication between the groups provides more opportunities for communication and interaction between the groups, and also enables the bullies in the group to learn to solve the problem in a more reasonable way. Bullies reflect on their daily behaviors and thoughts, so that group members can have a deeper understanding of themselves, learn how to deal with irrational thinking, figure out how to control and reduce irrational thinking, and learn how to rebuild rational thinking in group intervention. To fundamentally solve the extremist thoughts of the bully to lay a certain foundation of the course.
  Lesson Summary: Bullies in groups tend to be more actively involved in thinking and acting, and they have difficulty staying silent during interactions with others. Bullies like to “rhythm” and interfere with others' sharing, and tend to stray from the group's theme. Social workers need to remind and emphasize the working group's curriculum themes, as well as discipline issues in the curriculum. Because social workers need to spend a lot of time and energy on maintaining the theme and order, their ability to grasp the course schedule is relatively weak. In order to consolidate and practice the daily communication skills and methods of the working group in the teaching of the course, before the end of the course, the social workers assigned some assignments on the subject of the course, and shared their experience in the next class, so as to ensure that the team members understand the consequences of irrational thinking.
(4) Section 4 Working Group: Emotion Control and Interpersonal Communication Skills. See Table 6 for details.
  Course Analysis: The fourth part of the working group continues to focus on the issue of emotional control for bullies. The bullies in the group shifted from the resistance group to the acceptance and trust group. In this lesson, the task force shares several ways to calm down a bully in an irrational mood. In addition, through role-playing skills, the team members simulated the psychological activities they experienced in different environments, used “I” to express their thoughts every day, and strengthened their control over bad emotions. Let the bully represent the bullied, learn to put yourself in others' shoes, so as to achieve the thinking change of “putting yourself in others' shoes”, understand the harm of school bullying behavior, and reject bullying behavior from the heart.
  Course Summary: The content of the working group is very complete. In this course, the group members have learned how to calm down their emotions, get rid of the control of irrational emotions, experience the inner changes in the role so as to achieve the requirement of self-reflection. Bullies can actively participate in the interaction when they play other roles. Although some students with good face avoid interaction because they are afraid of making a fool of themselves, they finally complete the role playing through the persistence of social workers and the encouragement of their peers. Through communication and interaction, the team members' trust in the team and the friendship among them are enhanced. The group work is progressing smoothly, but there is a lack of negative emotion venting and mature emotion control system in the course. Therefore, we need to adopt appropriate incentive policies and reasonable material rewards, so that team members can more actively participate in the interactive activities of the working group.
(5) Section V Working Group: Behavior Attribution of Bullies. See Table 7 for details.
  Course Analysis: The subject of this course is the victim of bullying. First, ask the victim to share positive things in their life, as well as brave things in their daily life. According to the characteristics of the victims, the thinking mode of the victims when they encounter school bullying is analyzed from different perspectives. The teaching video “Know Yourself” and “Snack Fight” were shown. The victims were asked to describe the personality and mood of each character in the video, as well as the influence of their way of doing things. Share the bullying behavior and its characteristics in the video, so that they can get the opportunity to understand themselves in the communication, and lay the foundation for changing the bullying.
  Lesson Summary: When the victim joins the work group, the group members have no emotional involvement and are only attracted to the group when they watch a short film shown by the work group. The group members were relatively quiet and could not relax during the activity. Social workers must play ice-breaking games at the beginning of classes, as well as more interactive sessions. Make the team members who have been bullied have confidence in the working group, and more conducive to enhance the cohesion of the group.
(6) Section 6 Working Group: How to say no to school bullying in terms of behavior. See Table 8 for details.
  Course Analysis: This course is the last course of social work intervention, and also the last stage of school bullying management. The last class of the working group went smoothly and recorded the school bullying play performed by the whole group by means of photography and video. Members of the team create mementos by recording the scripts they create, produce and execute themselves into video clips. At the end of the stage play, the social workers arranged all the members to write a letter from the heart, put the letter in a time capsule and buried it under the tree on campus. Because of the active participation in the group, the team members unite, trust and encourage each other in the course, and finally learn to correctly understand campus bullying, how to reasonably control emotions and protect themselves. Finally, the staff shared the changes of all team members in this course, and encouraged and recognized all the students who participated in the working group.
  Summary: The working group has entered the final stage of intervening in school bullying. At this stage, the members have been able to independently recognize their responsibilities and unite with each other. Compared with the initial stage of the group, the group members' identity with the group and with each other has changed remarkably. During this period, the group members had a deeper understanding of school bullying in the development of group courses, and they also recognized the teaching methods of drama courses more. In the process of sharing and interacting with each class, social workers actively guide students' thinking direction and respond to students' questions. Actively guide the students to summarize their own learning results, so that they can flexibly use the interpersonal skills learned in the drama course in real life. In this for the intervention of social work, draw a perfect full stop.

**Table 3:** Working group activities and establishment of group relations.

**Number of sessions**	**Working group objective/working group activities content**	**Material**
Section I	Working group goal: the team members are familiar with each other, understand each other, establish the working group goal.	Sand table, white paper, marker pen, sand ware
	1. Introduction to staff and members	
	2. Use sand table and sand painting activities to organize group members' internal interaction and improve group members' sense of identity.	
	3. Determine the activity content and stage goal of the working group, and obtain the expected effect of the team members on the working group.	
	4. Establish the contract of the working group and the implementation standard of the activity	
	5. Notice in the next section.	

**Table 4:** Campus bullying identification course.

**Number of sections**	**Working group objective/working group activities content**	**Material**
Section 2	Working group goal: To enable all team members to identify what constitutes bullying and to distinguish the types of bullying.	Stool
	1. The warm-up game: Who's undercover	
	2. Discuss in pairs, put forward what you think about school bullying, and share your understanding of drama courses.	
	3. Play the school bullying movie “Teenage You”, share your impressions and introduce the bullying knowledge in it.	
	4. Pause some scenes in the movie, and analyze the type characteristics of such bullying behaviors.	
	5. Summary speech and the next section notice.	

**Table 5:** The unrational behavior courses for understanding the bullying self.

**Number of sessions**	**Working group objective/working group activities content**	**Material**
Section 3 (Bullies)	Task group goal: Make the bully aware of his irrational thoughts	Work notes
	1. Ask the bully in the group to share the irrational thoughts that they have on a daily basis and explain why.	
	2. Use the self-questioning method to ask the school bully some of his own irrational thoughts, do I have to do this? If I was injured, what should I do? And discuss new solutions with everyone.	
	3. Through some irrational behaviors in real life, this paper analyzes the thinking process of self rhetorical question through cases, and then puts forward a reasonable approach through discussion.	
	4. Make a summary speech and encourage the bullying participants to make a difference.	
	5. In the next section.	

**Table 6:** Emotional control and interpersonal skills.

**Number of sessions**	**Working group objective/working group activities content**	**Material**
Section IV (Bullies)	Working Group's goal: to enable school bullies to effectively control their emotions and acquire interpersonal skills.	Work notes, water pens
	1. In the emotional more excited, through effective skills to gradually calm down.	
	2. Role-playing is adopted to set up a specific social environment so that the bully can play a student who can solve his own emotional problems.	
	3. Express yourself in soothing, gentle, and polite language by introducing your ideas.	
	4. Summarize the speech and affirm the change of the bully's behavior.	

**Table 7:** Behavior lying of bullied behavior.

**Number of sessions**	**Working group objective/working group activities content**	**Material**
Section V (Bullying)	Working group goals: To understand the thoughts of the bullied, analyze the bully's situation and behavioral attributions.	Work notes, water pens
	1. The victim shares their positive energy of being brave in life.	
	2. Games: the right interpretation.	
	3. Examples are given to illustrate some thinking patterns and behavioral logics in the individual environment of different team members.	
	4. Conduct behavioral attribution questionnaire and boundary perception expression	
	5. Play teaching videos “Know Yourself” and “Battle for Snacks” to analyze the time, roles, emotions, ideas and influences in the videos.	
	6. How can I protect myself after sharing lessons.	
	7. Summarize the speech and encourage the victims to fight back and seek help.	

**Table 8:** Say no to bullying on campus.

**Number of sections**	**Working group objective/working group activities content**	**Material**
Section 6 (All team members)	Working Group Goal: Show how the bully's real thoughts can be expressed properly through role play, and how the bullied can say no in the face of school bullying.	Camera, camera, work notes, time capsule model
	1. Theater stage performance.	
	2. Each team member writes a letter from his heart, puts it in a time capsule, and buries it in the campus together.	
	3. The staff members share and summarize the changes of the team members in the activity.	
	4. Take photos to commemorate.	
	5. Finish the job.	

### Drama Course Effect Evaluation

At the end of the drama course, students participating in the drama course were asked to fill in the drama course effect self-scale, and the impact of drama course on school bullying was evaluated according to the proportion of students in the drama course. The results are shown in Table 9.

**Table 9:** Drama course effect self—scaletitle.

	**Number of people**	**Proportion (%)**
I can identify the types of bullying on campus.	6	100
I learned about drama classes	6	100
I can control my irrational thoughts	5	83.3
I've mastered interpersonal skills	6	100
I learned what to do when there is bullying on campus	6	100
I can handle my ideas in the right and reasonable way	4	66.6
I can lead by example and be a peace elite	5	83.3
I learned that escape or violence is not the right solution	6	100
I understand how unreasonable some of the past behavior, I will control or protect themselves.	6	100
I think drama course can effectively control campus bullying	4	66.6
I would like to continue to participate in the activities organized by the social working group	5	83.3

As shown in the table above, In the self-assessment, most of the team members are very clear about the meaning of campus bullying, and have a good understanding of the skills and knowledge of drama course. Through the drama course, the team members have a better understanding of the thinking process of the bully and the bullied, and learned to think in other places, which can improve the bullying behavior on campus. Thirty percent of the students think that they can solve problems with their own methods, which shows that the knowledge taught by the working group cannot be easily used in practice. But more than half of the team members clearly realized that violence did not solve the problem. And realize that violence is a kind of behavior that brings negative influence to the students around them. Only one member passed the scale with full marks. He thought that he could better understand himself through this group. It can also make other team members realize some of their own ideas and understand others' positions and behaviors by themselves and others. Most of the group members don't want to encourage their friends to join the working group, but they are happy to participate in other activities of the social workstation.

## Social Work Intervenes in Practical Difficulties

### Diversity of Types of School Bullying

There is a wide variety of types of bullying and bullying behavior is not fixed. School bullying can be divided into three main categories: the first is verbal bullying. Verbal bullying is a common phenomenon in daily life on campus. Abuse, personal attacks, nicknames and so on are all verbal bullying; The second category is behavioral bullying. Behavioral bullying means that the bully intimidate others for a long time and cause physical harm to others, such as beating, deliberately pushing, tripping, punching and kicking, or even intentionally harming others' economic interests and life and property safety. Use blackmail, isolation, defamation, and coercion to do something against one's will. The third category is cyberbullying. With the progress of science and technology, the Internet has become an indispensable “necessity” for people, and the phenomenon of bullying comes along with the Internet. There is less pressure and constraint in online communication, so teenagers are more likely to vent their negative emotions without any cost when using platforms such as online social media, such as maliciously slander others and make rumors. Even in school forums, post-bars and other environments, there is bullying both online and offline. Therefore, it can be seen that the role types of school bullying are diverse.

### Personal Cognitive Biases of School Bullying

Students' personal factors play an important role in the prevalence of campus bullying. The cognition between people directly affects people's behavior. From the perspective of cognitive behavior theory, among the three factors of emotion, cognition and behavior, cognition plays a role of neutralization and coordination in emotion and behavior. Understanding and observing the same things in many ways will lead to different behaviors. Different cognitive biases of the same behavior can also lead to this result. In terms of campus bullying, with the deepening of bullying, the bully becomes more and more bossy and arrogant; However, the bullied become more and more cowardly. Some people fight against Campus bullying, while others choose to avoid self comfort. People's different views on campus bullying are also due to cognitive bias. The bully usually pursues power and face, thinks that he can get the experience of maximizing ability in bullying, and pursues security and sense of belonging through personal authority; Those who are bullied are often timid, dare not face the bullying, dare not speak up. Lead to their own long-term threat and injury, and ultimately that resistance is useless. Obviously, these are caused by personal cognitive bias.

When social workers asked students, “What is bullying in your mind?”, the students will only one-sided answer: being beaten, coercion. Most students don't realize that verbal abuse, teasing and isolation are also bullying. In order to better solve and prevent the problem of school bullying, schools need to train students to have a correct understanding of school bullying. The task force's research includes the recognition of irrational thoughts and attribution of thought and behavioral outcomes, the use of therapy to correct emotions, change false cognitive biases, and thus transform the way school bullies and victims think and behave, and develop interpersonal skills and confidence. To empower young people to speak up and reject bullying.

### The Emergency Management Mechanism of School Bullying Is Not Perfect

As a social worker, the author went to a high school and proposed to set up a school bullying working group. However, after the school learned of the author's intention, the school did not agree to set up educational courses and working groups on the subject of “school bullying”. Later, after the author found social workers, the school decided to change the theme of the working group to “harmonious campus”. During the drama class of school bullying working group, the author observed the students in each class and found the occurrence of campus bullying. There was a slight bullying incident on campus, which was only handled by the teaching director alone. When the bullying on campus is serious, the head teacher will contact the parents to give oral education. The results show that schools have no ability to control campus bullying due to the imperfect emergency management system.

## Suggestions on Countermeasures for School Bullying

### Improve Teenagers' Perception of Bullying

Teenagers are in the special period of adolescence, which is the key stage of psychological development. Many times, teenagers' misunderstanding of things will lead to cognitive bias. If young people want to understand things without one sidedness, they must first understand themselves correctly and form a correct outlook on life and values, which is manifested in dignity, self-confidence and love. First, teenagers must know how to deal with interpersonal relationships, learn interpersonal skills, respect others, especially in middle school life, and learn to control irrational thoughts. When there is conflict with others, we should be able to deal with the problem rationally. We should not use violence and bullying to solve the contradiction. Second, strengthen students' consciousness of self-protection; When they are bullied, they know how to actively resist and take the right way to protect their own interests and security.

### Establish a Good Family Relationship and Educational Environment

Parents are children's first teachers, parents' behavior will affect the children's life. The difference of family relationship and family education mode has an imperceptible effect on the development of adolescent personality. Parents should set an example and guide and educate their children to abide by laws, regulations and moral norms. They should not take a rude, non-interference and intolerant attitude toward the difficulties and setbacks their children encounter in the process of growth. Be patient, responsible and careful to teach them. At the same time, family members should communicate with each other at any time to create a harmonious and equal family atmosphere and family relations, promote family harmony and enhance parent-child relationship. Parents must keep close contact with schools and social workers, keep an eye on the phenomenon of school bullying, understand their children's behavior and life in school, track their children's psychological state and correct their psychological differences, so as to eliminate school violence. It is necessary to correctly guide and educate teenagers in the way of interpersonal communication, enhance their awareness of self-protection and prevent the occurrence of school bullying.

### Improve the Emergency Management Mechanism of School Bullying

In the case of school bullying, schools must take the main responsibility. In order to prevent the occurrence of campus bullying, the campus should take the following measures to manage bullying incidents. First, the campus should use billboards, broadcasts, publicity boards, forums, official websites and other media to create a campus atmosphere of mutual assistance, friendship, respect for teachers and education. The second is to set up the legal education and mental health class for middle school students, so as to popularize the legal knowledge and establish the legal awareness, so that students can know, abide by and understand the law, do not use violence to threaten the safety of other people's lives and property, and do not use bullying to coerce others into harmful behaviors. To cultivate students' good psychological quality, learn to release bad emotions and pressure, and educate students to reduce bullying behavior on campus. Third, teachers should pay more attention to the phenomenon of school bullying. The phenomenon of school bullying is mostly caused by teachers' neglect of students and students' incomprehension of teachers. Therefore, teachers should be fair to every student and can't ignore them. Do not despise or discriminate against school bullying, maintain sensitive contact, timely intervention to the school and parents, education, care and protection of students. Fourth, schools must cooperate with social workers to hold regular class meetings to discuss the problem of school violence. For students who have all kinds of bullying behaviors on campus, social workers should guide them to follow up and observe or intervene, and use professional theoretical knowledge and skills to solve the problem of school bullying, so as to change their thoughts and behaviors in an all-round way.

### Construction of Social Workstations and Multiple Network Mutual Assistance Mechanisms

To reduce the incidence of school bullying, social workstations should combine school and network to establish multiple mutual assistance mechanisms. Through the linkage and mutual assistance of “social work, school, family, network, and community”, we should give full play to the advantages of all aspects and mobilize all forces to protect the victims of bullying to the maximum extent and curb the phenomenon of school bullying. Through the school, family, network, community and other platforms, provide advanced services, use professional social work methods, distribute school bullying brochures at the school gate, to promote and identify school bullying education; To prevent and respond to bullying, schools should work with social workers to provide students with regular training on how to deal with bullying on campus. To clarify teachers' responsibilities and pay more attention to students' physical and mental state, so as to provide strength for the elimination of school bullying; Social workers must maintain contact with parents and guide parents in educating and protecting children within the family, properly dealing with bullying and avoiding bullying behavior (29, 30).

## Conclusion

At present, the phenomenon of campus bullying is becoming more and more serious. School bullying has become an important problem faced by schools and society. Therefore, the education department and scholars are very concerned about the problem of campus bullying, and the state has constantly issued laws and regulations to combat campus bullying, which can prevent the spread of bullying to a certain extent. As a social worker, we have the obligation to use professional theory, professional methods and social work practice skills to intervene in the core of campus bullying, and to intervene and control campus bullying from the root.

As for the problem of campus bullying, this paper combines social work intervention with drama therapy theory. The purpose is to change teenagers' cognition of campus bullying, make teenagers really understand the root of campus bullying, and improve teenagers' ability to deal with campus bullying. At the same time, for the bullies, the drama class carried out by the working group has improved their self-confidence and communication skills; For bullies, the key is to learn to control bad emotions and change irrational thoughts. The essence of the work of the working group is not only to improve the self-development of the group members, but also to pay attention to the bullying behavior inside and outside the campus to prevent its spread. However, due to the lack of theoretical basis and practical experience of social work, campus bullying has not been completely and effectively solved. The efficiency of the working group on school bullying needs to be improved.

Therefore, it is very important to combat campus bullying from all aspects. This is not only from the perspective of social work intervention, but also from the perspective of school, family and government, so that young people have a good and healthy learning and growth environment. To lay a solid foundation for the construction of harmonious campus and harmonious society.

## Data Availability Statement

The original contributions presented in the study are included in the article/supplementary material, further inquiries can be directed to the corresponding author.

## Ethics Statement

Ethical review and approval was not required for the study on human participants in accordance with the local legislation and institutional requirements. Written informed consent from the participants' legal guardian/next of kin was not required to participate in this study in accordance with the national legislation and the institutional requirements.

## Author Contributions

JZ conceived the research, designed, and drafted the manuscript.

## Conflict of Interest

The author declares that the research was conducted in the absence of any commercial or financial relationships that could be construed as a potential conflict of interest.

## Publisher's Note

All claims expressed in this article are solely those of the authors and do not necessarily represent those of their affiliated organizations, or those of the publisher, the editors and the reviewers. Any product that may be evaluated in this article, or claim that may be made by its manufacturer, is not guaranteed or endorsed by the publisher.
